# Long-term reduction of T-cell intracellular antigens leads to increased beta-actin expression

**DOI:** 10.1186/1476-4598-13-90

**Published:** 2014-04-27

**Authors:** Isabel Carrascoso, Carmen Sánchez-Jiménez, José M Izquierdo

**Affiliations:** 1 Centro de Biología Molecular ‘Severo Ochoa’, Consejo Superior de Investigaciones Científicas, Universidad Autónoma de Madrid (CSIC/UAM), C/Nicolás Cabrera 1, Cantoblanco, DP 28049 Madrid, Spain

**Keywords:** TIA1, TIAR, HuR, ACTB, Gene expression

## Abstract

**Background:**

The permanent down-regulated expression of T-cell intracellular antigen (TIA) proteins in HeLa cells improves cytoskeleton-mediated functions such as cell proliferation and tumor growth.

**Methods:**

Making use of human and mouse cells with knocked down/out expression of T-cell intracellular antigen 1 (TIA1) and/or TIA1 related/like (TIAR/TIAL1) proteins and classical RNA (e.g. reverse transcription-quantitative polymerase chain reaction, polysomal profiling analysis using sucrose gradients, immunoblotting, immunoprecipitation, electrophoretic mobility shift assays, ultraviolet light crosslinking and poly (A+) test analysis) and cellular (e.g. immunofluorescence microscopy and quimeric mRNA transfections) biology methods, we have analyzed the regulatory role of TIA proteins in the post-transcriptional modulation of beta-actin (ACTB) mRNA.

**Results:**

Our observations show that the acquisition of above cellular capacities is concomitant with increased expression levels of the actin beta subunit (ACTB) protein. Regulating TIA abundance does not modify ACTB mRNA levels, however, an increase of ACTB mRNA translation is observed. This regulatory capacity of TIA proteins is linked to the ACTB mRNA 3′-untranslated region (3′-UTR), where these proteins could function as RNA binding proteins. The expression of GFP from a chimeric reporter containing human ΑCΤΒ 3′-UTR recapitulates the translational control found by the endogenous ACTB mRNA in the absence of TIA proteins. Additionally, murine embryonic fibroblasts (MEF) knocked out for TIA1 rise mouse ACTB protein expression compared to the controls. Once again steady-state levels of mouse ACTB mRNA remained unchanged.

**Conclusions:**

Collectively, these results suggest that TIA proteins can function as long-term regulators of the ACTB mRNA metabolism in mouse and human cells.

## Background

Gene expression in higher eukaryotes is a multilayer process involving several complex processes, such as the transcription, splicing, translation and turnover of RNAs and proteins. It is now well-established that each step of this informative circuit is modulated by gene-regulatory events. So far each individual process has been strongly studied, but little is known about how the combined effect of all regulatory events shapes gene expression by multifunctional proteins. Thus, the fundamental question of how genomic and transcriptomic information is processed at different levels in response to dynamic regulatory changes and environmental stresses to obtain specific proteome diversity have therefore remained unanswered.

The T-cell intracellular antigen 1 (TIA1) and the TIA1-related (TIAR) proteins are multifunctional modulators at different layers of the gene expression regulatory flux and a good candidates to carry out these studies. These proteins are essential cellular components during early development because mice lacking TIA1 or TIAR as well as over-expressing TIAR, show high rates of embryonic lethality [1-3]. They can interact with DNA and RNA polymerase II (RNA pol II) to modulate the transcriptional rates of genes that encode functional proteins and RNAs [4-8]. TIA proteins are also splicing factors regulating around the 10% of pre-mRNAs splicing events occurring in the human genome [9]. These splicing regulators promote the U1 snRNP recruitment of the spliceosome machinery to splice constitutive and alternative exons containing introns with U-rich stretches adjacent to the 5′ splice sites [9-14].

In the cytoplasm, these proteins have dual roles in promoting stability and/or repressing translation of mRNAs involved in apoptotic and inflammatory pathways [1,8,15-17]. This capacity is associated to their binding at the 5′ and 3′-untranslated regions (UTRs) of the mRNAs containing U-, AU- and C-rich sequence contexts [7,9,18-22]. From a (patho-) physiological viewpoint, these regulators have been involved in the development of main biological processes and complex cellular responses, such as apoptosis, inflammation, virus-mediated infections, environmental stress, embryogenesis and tumorigenesis [1-3,8,23-27]. Although the roles of TIA proteins in key processes as, for example, inflammation, environmental stress and viral infections are well established, its effects on cell proliferation and tumor growth remain elusive.

To shed light on the potential role of TIA proteins in regulating proliferative responses, we used RNA interference strategy to knock down TIA expression in HeLa cells. We provide evidence that TIA-knocked down HeLa cells can enhance ACTB protein expression, accompanied by the ability to assemble actin stress fibers. This observation reveals a functional link at long-term between TIA proteins and the formation and remodeling of actin-based cytoskeletal structures. Given that TIA proteins can be associated with ACTB mRNA through its 3′-UTR, we show that the absence of TIA proteins facilitates the translation of a chimeric mRNA containing the 3′-UTR of the human ACTB mRNA. Mouse embryonic fibroblasts knocked out for TIA1 resulted in an increase in ACTB protein expression. Our observations suggest that the TIA proteins can play a regulatory role in post-transcriptional modulation of ACTB mRNA.

## Results

### Stable silencing of TIA proteins leads to an increase in expression of β-actin protein

**Figure 1:**
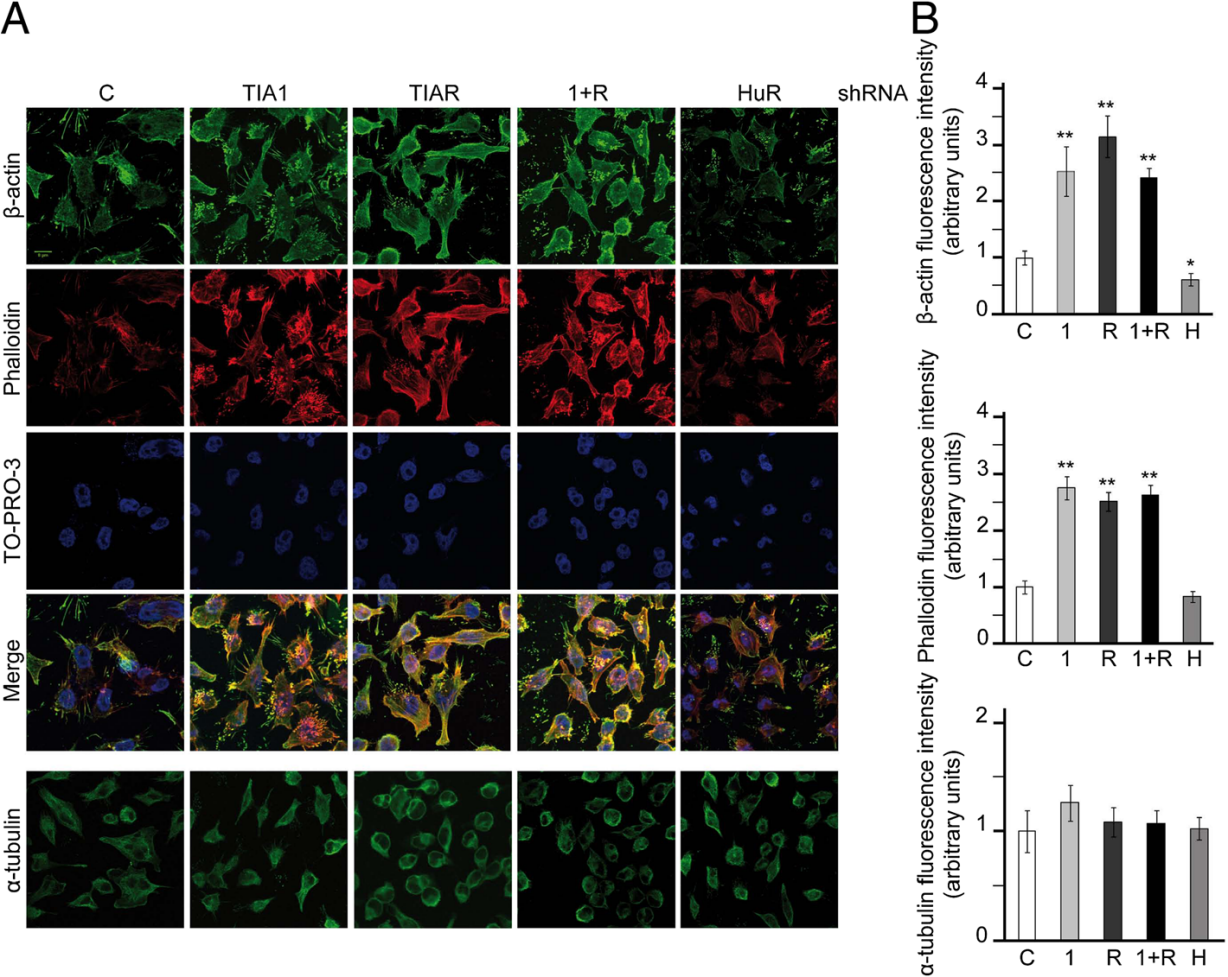
**Stable TIA knockdown leads to an increased expression of beta-actin protein. (A)** Exponentially growing HeLa cells stably transfected with control, TIA1, TIAR, TIA1 plus TIAR (1 + R) and HuR shRNAs were stained with anti-β-actin and anti-α-tubulin antibodies as well as incubated with phalloidin-TRITC and TO-PRO-3 reagents. The post-stained HeLa cells were visualized by fluorescence microscopy. **(B)** The fluorescence intensity of β-actin, phalloidin and α-tubulin were quantified from at least 30 different images per sample using ImageJ software. These quantifications are represented by histograms where the white, lightgray, darkgray, black and gray bars correspond to the control, TIA1, TIAR, TIA1 plus TIAR (1 + R) and HuR samples, respectively. The represented values were normalized and expressed relative to control (c), whose value is fixed arbitrarily to 1, and are means ± SEM (n = 30; *P < 0.05; **P < 0.01). The scale bar shows 8 μm.

Previous results revealed that the reduction of TIA proteins by stable RNA interference using specific short hairpin RNAs in HeLa cells triggers cytoskeleton-based functions, such as cell proliferation, migration and invasion [27]. These cellular phenotypes are ensured by a solid network of cytoplasmic stress fibers that connect different points at the cellular edges, leading to the maintenance of cell-cell or cell-extracellular matrix contacts [28]. Using the TIA-knocked down HeLa cells described above ([27] and Additional file 1: Figure S1), we investigated the effect of the reduction of TIA1 and/or TIAR proteins on the β-actin expression, a major component of cytoskeleton-based functions. HeLa cells were fixed and processed for indirect immunofluorescence with specific antibodies against TIA1, TIAR, HuR, β-actin and α-tubulin proteins, respectively, together with phalloidin to visualize stress fibers that are represented by polymerized F-actin proteins (Figure 1 and Additional file 1: Figure S1). The results of fluorescence intensity showed that the expression levels of β-actin protein and stress fibers were increased 2-3-fold in the absence of TIA1 and/or TIAR proteins (Figure 1A and B). By contrast, the expression of β-actin protein and stress fibers in HeLa cells was slightly down-regulated in the absence of HuR in agreement with previous data [29]. No significant differences were observed for α-tubulin protein expression between the experimental conditions examined. Collectively, these observations show the impact of TIA proteins in β-actin expression.

### The knockdown of TIA proteins increases the expression of β-actin protein without affecting the steady-state level of its mRNA

**Figure 2:**
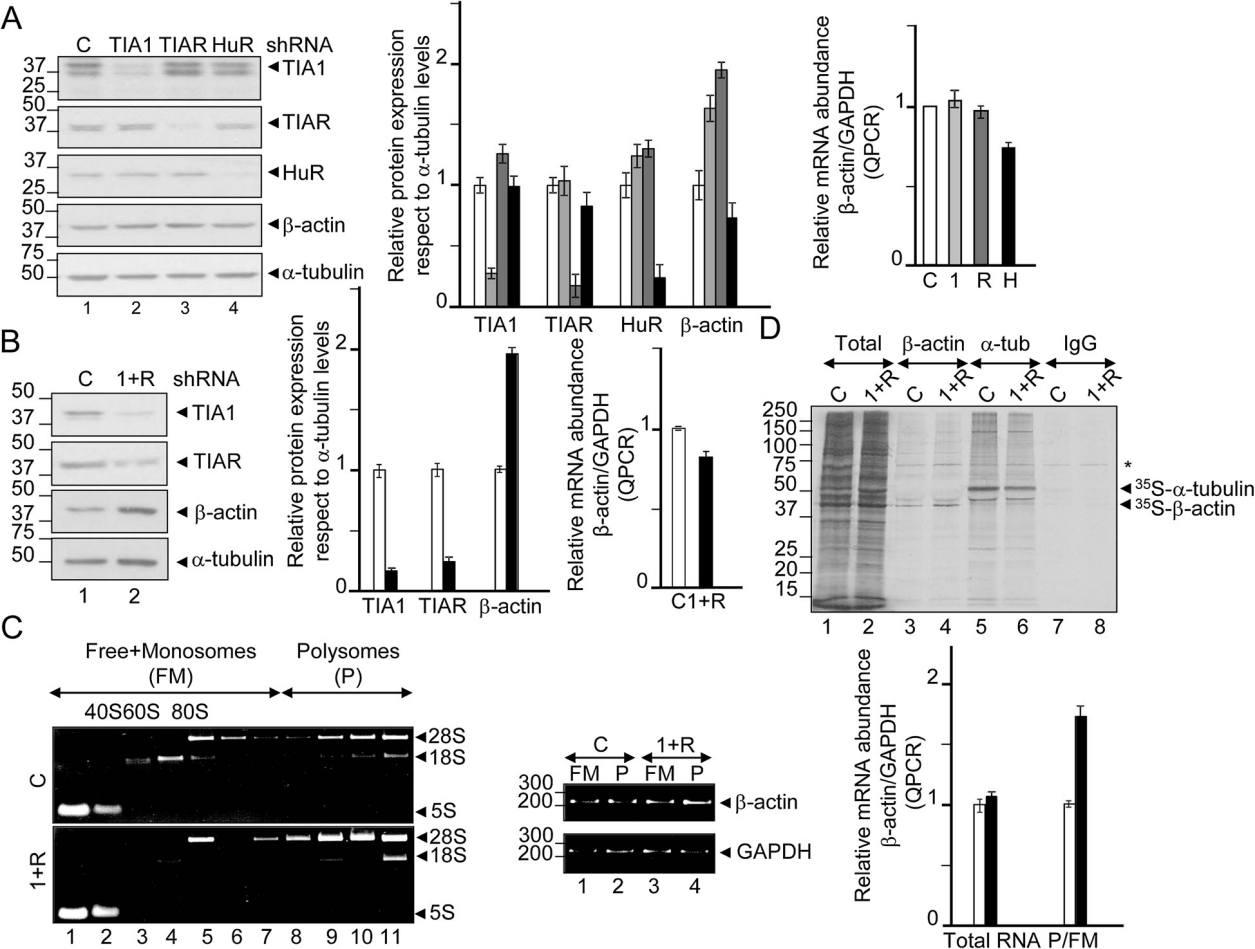
**Permanent TIA depletion improves β-actin mRNA translation. (A and B)** Western blotting and reverse transcription-real time PCR (RT-QPCR) analysis showing knockdown efficiencies of control (c, white bars), TIA1 (1, lightgray bars), TIAR (R, darkgray bars), TIA1 plus TIAR (1 + R, black bars) and HuR (H, gray bars) and their effects on β-actin protein expression and mRNA levels. **(C)** Polysome profiling analysis using sucrose gradients from control (c, white bars) and TIA1 plus TIAR (1 + R, black bars)-knocked down HeLa cells. The quality of polysome preparation was verified by electrophoresis analysis in TBE-2% agarose gels of RNA content in each fraction. Ribosomal 5S, 18S and 28S bands are shown. Density distribution of relative translational efficiencies of β-actin and GAPDH mRNAs was verified by semiquantitative and QPCR analysis, respectively, in fraction pools identified as free-monosomes (FM) (fractions 1-7) and polysomes (fractions 8-11). Data are the mean ± SEM from two independent analysis. The represented values **(A-C)** were normalized and are expressed relative to control (c), whose value is fixed arbitrarily to 1. **(D)** Nascent translation of total, β-actin and α-tubulin proteins was determined by incubation of control (c) and TIA1 + TIAR (1 + R)-silenced HeLa cells in the presence of ^35^S-methionine/cystein mix. After immunoprecipitation using anti-β-actin, anti-α-tubulin and IgG (negative control) antibodies, the newly translated proteins were visualized by 10% SDS-PAGE and autoradiography. In all indicated cases, molecular weight markers for protein or DNA and the identities of proteins and amplified PCR products are shown.

It is possible that the role of TIA proteins in cell behavior is mediated by its ability to regulate the metabolism of β-actin mRNA. As shown in Figure 2, we observed a significant increase (2-fold) in the levels of β-actin protein by western blot analysis in TIA1 and TIAR-knocked down HeLa cells compared to controls (Figure 2A, compare lane 1 with 2 and 3) and the HuR-silenced HeLa cells (Figure 2A, compare lane 1 with 4). These increases were not accompanied by similar changes at the steady-state levels of the β-actin mRNA (Figure 2A). Given that similar results were obtained with TIA1 plus TIAR-knocked down HeLa cells (Figure 2B), we concluded that TIA proteins can regulate the expression of the β-actin mRNA at the post-transcriptional level. Likewise, endogenous β-actin mRNA levels in HeLa cells did not change after stable silencing of TIA proteins, while the expression of the encoded β-actin protein increased markedly without apparent changes in β-actin mRNA stability. These observations suggest that the TIA proteins could be modulating its translation. In this regard, evidence that TIA proteins modulate translation of the β-actin mRNA was obtained by polysomal profiling analysis. Cytoplasmic extracts from control and TIA-knocked down HeLa cells were fractionated through sucrose gradient, with the lightest components sedimenting at the top (fractions 1 and 2), small (40S) and large (60S) ribosomal subunits and monosomes (80S) in fractions 3-6, and progressively larger polysomes in fractions 7-11 (Figure 2C). The distribution of the β-actin mRNA versus GAPDH mRNA, known as housekeeping gene, was measured by grouping fractions in free plus monosomes (FM) (fractions 1-7) and in polysomes (P) (fractions 8-11). The pools of the RNAs in FM and P fractions were quantified by semiquantitative reverse transcription and polymerase chain reaction (RT-PCR) as well as by real time RT-PCR in both total RNA and gradient fractions (Figure 2C). The results show an enrichment of the β-actin/GAPDH ratio (2-fold) in polysomes versus free + monosomes fractions in TIA-silenced HeLa cells. Additional evidence that TIA proteins modulated β-actin translation was gained from nascent translation analysis. We determined the incorporation of ^35^S-methionine and -cystein into newly synthesized β-actin during a brief time period (30 min), immediately followed by immunoprecipitation using anti-β-actin monoclonal antibody. This experiment reveals an enhanced translation of β-actin in the TIA-knocked down HeLa cells, whereas translation of the α-tubulin protein was unchanged between the two groups (Figure 2D). Taken together, these results suggest that TIA proteins regulate β-actin production by modulating β-actin mRNA translation.

### TIA proteins associate with β-actin mRNA through 3′-untranslated region

**Figure 3:**
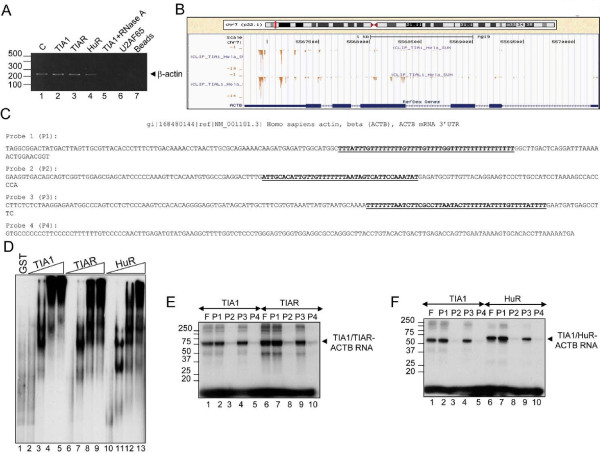
**TIA proteins bind to the human β-actin mRNA 3′-UTR. (A)** TIA and HuR proteins interact with β-actin mRNA. HeLa extracts were incubated with anti-TIA1 (in the absence or presence of RNase A), anti-TIAR, anti-HuR and anti-U2AF65 antibodies. Immunoprecipitated RNAs were isolated and quantified the β-actin mRNA levels by semiquantitative RT-PCR. The resulting PCR products were analyzed by 2% agarose-electrophoresis. In lane 1 (indicated as c above legend), 1/10 of the HeLa extract used in immunoprecipitation assays was amplified by RT-PCR. As a negative control, the beads were used (lane 7). **(B)** RNA map of TIA proteins on human β-actin (ACTB) gene by iCLIP analysis. Crosslinking sites of TIA1 and TIAR on the human β-actin gene in HeLa cells. The bar graph shows the number of cDNAs that identifies each crosslinking site [9]. Note that the highest density of crosslinking sites of the TIA1 and TIAR proteins is located at the 3′-UTR of the β-actin mRNA (for more details see Additional file 2: Figure S2). **(C)** Nucleotide sequence of the 3′-UTR of the human β-actin mRNA (NM_001101.3). The full-length sequence of the β-actin 3′-UTR was divided to generate four probes. In the probes 1 and 3, the major sites bind TIA proteinswere underlined. **(D)** RNA band shift assays of TIA proteins to β-actin 3′-UTR. Electrophoretic mobility shift assays were performed incubating GST (500 ng), GST-TIA1, GST-TIAR and MBP-HuR (0, 10, 100, 500 and 1000 ng) proteins with ^32^P-labeled β-actin 3′-UTR RNA. **(E and F)** Ultraviolet crosslinking assays illustrating the direct interaction between TIA1, TIAR or HuR proteins and β-actin 3′-UTR RNA probes. The F and P1-P4 legends correspond to full-length β-actin 3′-UTR RNA and the four β-actin RNA probes indicated in **C**, respectively. Molecular weight markers and the identities of protein-RNA complexes are shown.

Next, we tested the interaction of TIA proteins with the mRNA encoding human β-actin. By ribonucleoprotein immunoprecipitation analysis from cytoplasmic extracts from HeLa cells, β-actin mRNA was significantly enriched in TIA1, TIAR and HuR immunoprecipitation compared with TIA1 plus RNase A treatment, U2AF65 and the beads (Figure 3A). To find out the putative RNA sequences of binding we used the information maps for TIA proteins generated by Ule’s group using *in vivo* ultraviolet cross-linking and immunoprecipitation (iCLIP) analysis [9]. The RNA map of TIA proteins for ACTB pre-mRNA suggests that the prevalent RNA region to bind these proteins could be located on the 3′-untranslated region (UTR) of the β-actin mRNA (Figure 3B and Additional file 2: Figure S2). Before to test this possibility, we analyzed the putative heterogeneity of this RNA sequence using several different strategies, such as RT-PCR, RACE and RLC combined with poly (A) test (PAT) analysis (Additional file 3: Figure S3). The results suggested that the 3′-UTR region of human β-actin mRNA between two examined groups of HeLa cell lines is identical (Additional file 3: Figure S3). It was previously shown that β-actin mRNA associates with the HuR protein with high affinity and specificity on 3′-UTR RNA sequences of this messenger [29]. To determine whether the TIA1 and TIAR proteins bind also this region of β-actin mRNA, we performed electrophoretic mobility shift assays using the full-length 3′-UTR radiolabeled probe of β-actin mRNA and the recombinant GST-tagged TIA1 and TIAR proteins as well as recombinant MBP-tagged HuR protein. When these proteins were incubated with the full-length RNA probe, similar RNA-binding complexes were observed (Figure 3D, lanes 2-13). However, GST alone did not form any complex with this probe (Figure 3D, lane 1). These results suggest that TIA proteins, like HuR protein, can bind human 3′-UTR RNA of β-actin mRNA with a similar affinity. To confirm the iCLIP data (Figure 3B) and to know if the individual sequences on β-actin 3′-UTR (Figure 3C, see U-rich stretches underlined on probes 1-3) are the prevalent sites where TIA proteins interact with this RNA, the full-length β-actin mRNA 3′-UTR was divided in four portions (probes 1-4 in Figure 3E and F). When the four probes were incubated with recombinant versions of TIA1, TIAR and HuR proteins, irradiated with ultraviolet light and treated with RNase A, an RNA-binding complex was observed with the probes 1 and 3 but not with the others (Figure 3E and F). All these data, combined with the previous TIA-iCLIP findings, suggested that TIA proteins could interact with the 3′-UTR of β-actin mRNA.

**Figure 4:**
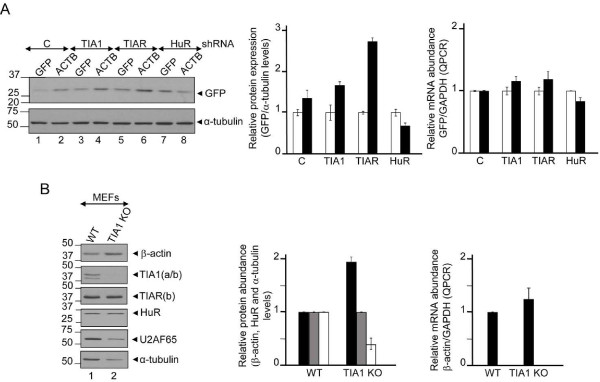
**The ablation of TIA1 gene in murine embryonic fibroblasts increases the expression of mouse β-actin protein without affecting the levels of mRNA. (A)** A chimeric GFP RNA containing β-actin 3′-UTR reproduces the regulation of the endogenous human β-actin mRNA in control (c), TIA1, TIAR and HuR-knocked down HeLa cells. The relative expression levels of GFP protein versus endogenous α-tubulin levels as well as the levels of reporter GFP mRNAs versus endogenous GAPDH mRNA were quantified by western blot analysis and QPCR, respectively. The quantifications are represented by histograms where white and black bars correspond to the GFP protein/RNA expression from GFP and GFP-β-actin 3′-UTR transfected plasmids, respectively. **(B)** Effect of the disruption of mouse TIA1 gene on β-actin expression in murine embryonic fibroblasts (MEF). Protein extracts and cytoplasmic RNAs from normal murine embryonic fibroblasts (WT MEFs) or knocked out for TIA1 (TIA1 KO MEFs) were purified and processed for western blot analysis with anti-β-actin, anti-TIA1, anti-TIAR, anti-HuR, anti-U2AF65 and anti-α-tubulin antibodies, or for QPCR analysis with mouse β-actin and GAPDH specific-mRNA primers, respectively. Molecular weight markers for protein (kDa) are indicated on the left. The identities of protein bands are indicated on the right by arrowheads. The represented values by histograms were normalized and are expressed relative to control (c), whose value is fixed arbitrarily to 1.

To test if the interaction of TIA proteins with the β-actin 3′-UTR was functional, we prepared reporter constructs derived from plasmid pEGFP-C1 in which the entire human β-actin 3′-UTR was inserted after of the GFP coding region. The corresponding control pEGFP-C1 was generated by direct mutagenesis inserting a stop codon to produce the same GFP protein above. After transfections of each reporter construct into HeLa cells expressing control (empty shRNA) or knocked down TIA and HuR proteins (TIA1, TIAR or HuR shRNAs), GFP expression was assessed by western blotting (Figure 4A). As observed, the knockdown of TIA proteins selectively increased GFP production in pEGFP-C1-β-actin 3′-UTR, but not significantly in controls. This increase in GFP protein did not arise from changes in GFP mRNA (Figure 4A). In the same vein, to test if the interactions of TIAR and HuR with the β-actin 3′-UTR were functional, we co-transfected the previous pEGFP-C1 and pEGFP-C1-β-actin 3′-UTR constructs in the absence or presence of TIAR- and HuR-tagged pEGFP-C1 plasmids (Additional file 4: Figure S4). As observed, the over-expression of TIAR selectively decreased GFP production, but not in the control (empty plasmid) and HuR reporter groups (Additional file 4: Figure S4, compare lanes 3 and 4 with 1, 2, 5 and 6). Collectively, these results suggest that TIA and HuR can regulate translation and/or stability of β-actin mRNA.

### The disruption of TIA1 gene in murine embryonic fibroblasts leads to an increase in the expression of β-actin protein without changes in the mRNA levels

The above results support a role for TIA proteins in the post-transcriptional modulation of human β-actin mRNA expression exerted through its 3′-UTR. Given the high degree of conservation between human and mouse 3′-UTR sequences (Additional file 5: Figure S5), we decided to examine the potential effects of TIA1 protein on β-actin protein expression as well as the degree of regulatory conservation of post-transcriptional mechanisms between men and mice. For this purpose, endogenous β-actin protein and mRNA levels were compared in two fibroblast cell lines of mouse [1]: (i) a wild-type mouse fibroblast cell line (WT MEF) and (ii) a mutant cell line in which the two copies of the TIA1 gene had been knocked out (TIA1 KO MEF). Western blot analysis showed that β-actin expression level is increased approximately 2-fold in TIA1 KO MEF compared to WT MEF (Figure 4B); despite the levels of α-tubulin and U2AF65 protein being lower in TIA1 KO MEF cells than in the wild-type culture. No differences in the expression level of HuR protein was detected in the mouse cell types examined (Figure 4B). The augmented expression of β-actin protein in TIA1 KO MEF cells is not due to the state-steady levels of β-actin mRNA because similar abundance of β-actin mRNAs was found by quantitative RT-PCR in the two mouse cell lines (Figure 4B). Thus, MEFs with TIA gene disruption show an increased level of β-actin as previously observed in HeLa cells. The fact that TIA proteins exert such function in both HeLa and MEFs suggests that they display a master regulator function in β-actin expression.

## Discussion

Our results support that a sustained knockdown of TIA1 and TIAR proteins in HeLa cells and the gene ablation of TIA1 in mouse embryonic fibroblasts leads to increased expression of β-actin protein without significant changes at the steady-state levels of the β-actin mRNA. These observations are consistent with a translational modulation of β-actin mRNA and the binding of TIA proteins on the β-actin mRNA through its 3′-UTR. This finding is coherent with the RNA map (iCLIP analysis) of interactions among β-actin mRNA and TIA1/TIAR proteins described by Ule’s group [9]. By contrast, it was unexpected to find that TIA proteins may be involved in the translation of the β-actin mRNA, since transient knockdown of these regulators did not affect its translational efficiency [21,22] and Additional file 6: Figure S6]. Thus, it is possible that the binding to long U-rich stretches both in TIA and HuR proteins could repress and/or stabilize β-actin mRNA in response to dynamic behaviors of gene regulatory networks associated with environmental stresses. This association might be stable and compensatory throughout during the life span of the cell, but it could be altered by the permanent deficiency of some specific RNA binding proteins and/or RNAs as, for example, microRNAs and/or non-coding RNAs.

The actin proteins in the cell of higher eukaryotes are encoded by six individual genes [30]. The global features of these protein products are their ubiquitous distribution, stability and high concentration. The expression of actin genes is regulated at the transcriptional level [31]. However, regulatory post-transcriptional events such as the cellular localization, stability and translation of their mRNAs affect where and how these proteins will be synthesized in the cell [32-39]. The specific localization of each protein isoform corresponds to the precise and exact distribution of their mRNAs which are targeted for translation [33-35]. The selective localization, stability and/or translation of the β-actin mRNAs is regulated by the collaboration of a specific bipartite sequence in the 3′-UTR and the zip code-binding protein (ZBP) [36-39]. This regulatory event is a paradigm to understand the relevance of localization, movement, motility of cellular mRNAs to generate selective distribution, targeting and expression of proteins in order to promote functional heterogeneity to participate in the formation and maintenance of important cellular structures such as extracellular matrix, stress fibers and lamellipodia [40-42].

Stability/turnover and translation of eukaryotic mRNAs are two sides of the same coin from a strictly regulatory viewpoint of the expression gene at the post-transcriptional level. Given that TIA proteins, like HuR protein, are known to stabilize and/or to translational repress/activate mainly short-lived mRNAs (for example mRNAs encoding cytokines, lymphokines, protooncogenes and growth factors) that are required for cell differentiation/proliferation, inflammation or apoptosis [1,8,9,15-17]. These cellular mRNAs are quickly translated and/or degraded because it is crucial a limited concentration of their products to protect the cell from undesirable effects, as for example an uncontrolled growth. The TIA-iCLIP analysis in HeLa cells previously showed that highest enrichment density of cDNAs were seen in introns and 3′-UTRs [9]. These observations are consistent with the role of TIA proteins in pre-mRNA splicing, stability and/or translational regulation via binding U-, C- and AU-rich sequence elements located at the 5′ spliced sites and 3′-UTRs, respectively [1,7,9,14,15,17,19,20]. It is interesting that no overlap was observed between gene targets reported to be regulate by TIA1 at the translational level [19] and genes predicted to be regulated by TIA1/TIAR at the alternative pre-mRNA splicing level [14]. This observation suggests that TIA proteins could be regulating distinct subsets of genes at the splicing and translational levels. The TIA-iCLIP data have expanded the total number of post-transcriptional events and associated gene functions that could be predicted to be regulated by TIA proteins. Thus, the estimated frequency of cellular events regulated by TIA1/TIAR via specific-sequences motifs might be approximately 20-30% [9,14].

The mechanism of TIA-induced translational repression has been partially characterized for conditions of environmental stress such as oxidation and nutrient deprivation responses. For example, under oxidative conditions eIF2α phosphorylation inhibits translation initiation and TIA proteins contribute to the assembly of unproductive translation pre-initiation complexes with the mRNA to stress granules [43]. When cellular scenario is upon amino acid starvation, TIA proteins assemble onto the 5′ end of the 5′-terminal oligopyrimidine tracts (5′-TOP) mRNAs and arrest translation at the initiation step. The TIA-dependent 5′-TOP mRNA translation repression implies polysomal release and accumulation in stress granules [21]. This repressor activity requires starvation-mediated activation of the GCN2 (general control non-derepressible 2) kinase, which could be activated by the accumulation of unchanged tRNAs, and inactivation of the mTOR (mammalian target of rapamycin) signalling pathway [21], although this is a controversial issue [22]. These observations could explain limitedly how large networks of cellular RNAs encoding protein biosynthesis factors can be post-transcriptional co-regulated in response to nutrient availability by specific RNA binding proteins as TIA proteins.

We have previously reported that TIA-deficient HeLa cell lines produced larger and faster-growing tumors following injection in nude mice [27]. Further, we have described that TIA protein expression levels decrease in many cancer cell lines [44] and some human tumor types [27], suggesting that TIA proteins might play a relevant role as suppressor genes of cell proliferation and tumor growth. It is now well established that a main feature of cancer cells is uncontrolled proliferation. An increased expression level of β-actin protein is required for relevant biological processes involving from cell shape, growth to the motility, because β-actin is a major component of the cytoskeleton in non-muscle cells [28,42]. Therefore, this raises the possibility that the permanent reduction of the TIA proteins in several cancer cells improves the expression of the β-actin protein, which in turn modifies cytoskeleton organization and cellular functions/phenotypes.

## Conclusions

The aim of this study was to investigate how the permanent down-regulation of the T-cell intracellular antigens (TIA1 and TIAR) leads to the uncontrolled cellular growth phenotypes. Consistent data are provided demonstrating that these effects are due, at least in part, to the increased expression of the β-actin (ACTB) that, consequently, promotes the assembly of cytoskeletal stress fibers. Given that the steady-state levels of the ACTB mRNA remained unchanged under these conditions, the results suggested that TIA proteins may be regulating the translation of the ACTB mRNA. By assessing the steady-state levels of the ACTB mRNA on monosomal and polysomal fractions purified by sucrose gradients and by immunoprecipitating of metabolically-labeled ACTB protein in control and TIA-down-regulated HeLa cells, TIA proteins can modulate the translation of the ACTB mRNA. Immunoprecipitation of ribonucleoprotein complexes coupled to RT-PCR analysis, electrophoretic mobility gel shift assays and quimeric RNA transfections pointed out the potential binding of TIA1/TIAR proteins with ACTB mRNA in a 3′-UTR-dependent manner. Additionally, the expression of the ACTB protein was greater in mouse embryonic fibroblasts (MEF) knocked-out for TIA1 than control MEF. Taken together, these observations support that sustained reduction of TIA proteins enhances expression of ACTB protein, suggesting a role for TIA proteins as repressors of ACTB mRNA translation.

## Methods

### Cell cultures, plasmids and recombinant proteins

Adherent HeLa cell lines expressing stably empty (control), TIA1 and/or TIAR, and HuR shRNA-pSUPER constructs were cultured under standard conditions as previously reported [27]. Murine embryonic fibroblasts (MEFs) wild-type and knocked out for TIA1 were generated and grown as described [1,45]. The 3′-UTR sequence from the human β-actin mRNA was generated by RT-PCR from cytoplasmic RNA or by PCR from genomic DNA purified of HeLa cells by using reverse transcriptase (Promega) and/or Pfu Turbo DNA polymerase (Stratagene), respectively. The construct used to test the translational efficiency of human β-actin 3′-UTR RNA was generated by subcloning the corresponding PCR product to the carboxy-terminal part of GFP using vector pEGFP-C1 (Clontech). In this construct, as well as control GFP-plasmid, was introduced a stop codon by direct mutagenesis expressing a truncated version of GFP lacking the last 18 amino acids at the carboxy terminal end. The sequences of all constructs were verified by automated DNA sequencing. Recombinant GST, TIA1, TIAR, and HuR proteins were expressed in *Escherichia coli*, purified as glutathione-*S*-transferase (GST) or maltose-binding protein (MBP) fusions using glutathione-Sepharose (Sigma) or amylose resin (BioLabs), respectively, and stored in buffer D (20 mM Hepes, pH 7.6, 20% glycerol, 0.2 mM EDTA, 1 mM DTT, and 0.01% NP-40) plus 0.1 M KCl at -70°C until used [46].

### Fluorescence microscopy

The different HeLa cell lines were grown for 24 h on coverslips and processed as previously described [47]. Endogenous TIA1/TIAR/HuR/β-actin/α-tubulin-antibody complexes, TO-PRO-3, or phalloidin-stained proteins were viewed by confocal microscopy. The quantification of relative fluorescence intensity corresponding to the used molecular markers was carried out using ImageJ software. The results shown in the corresponding histograms are average of at least independent 30 images for each condition examined.

### Protein analysis and RNA isolation

Whole-cell HeLa extracts were prepared by resuspending the cells in lysis buffer (50 mM Tris–HCl, pH 8.0, 140 mM NaCl, 1.5 mM MgCl_2_, 0.5% Nonidet P-40 plus a cocktail of protease inhibitors), freeze-thawing 3 times, and centrifugation at 10,000 rpm for 10 min in a microfuge at 4°C. The supernatants were recovered and stored at -70°C. Protein concentration was determined with the Bradford reagent (Bio-Rad). Western blots were carried out using nylon membranes and the following antibodies: anti-β-actin (AC-74, Sigma), anti-TIA-1 (C-20, Santa Cruz Biotechnology), anti-TIAR (C-18, Santa Cruz Biotechnology), anti-HuR (3A2, Santa Cruz Biotechnology), anti-GFP (JL-8, BD Biosciences Clontech), and anti-α-tubulin (B-5-1-2, Sigma). The blots were developed using ECL reagent (Amersham Pharmacia Biotech). Cytoplasmic RNAs were prepared using the RNeasy kit (Qiagen). Cytoplasmic RNA was quantified by optical density at 260 nm and treated with RNase-free DNase (Promega). For QPCR, an aliquot (5 ng) of the cDNAs and 0.5 μM of each primer together with Power Sybr Green PCR Master mix (Applied Biosystems) were used following the protocol: 10 min at 95°C followed by 20–40 cycles of denaturation (15 s at 95°C) and annealing–elongation (1 min at 60°C) with fluorescence acquisition at 60°C. A melting curve (15 s at 95°C, 15 s at 60°C, and 15 s at 95°C) with fluorescence acquisition at 60–95°C was included in each qPCR. An aliquot (1/10) of the cDNAs was amplified by QPCR using, GFP- and GAPDH-specific oligonucleotide pairs [8]. The amplification efficiency of each primer was empirically determined and applied to the relative quantification of the data using qbase software (http://medgen.ugent.be/qbase/). In semiquantitative RT-PCR analysis, RNA was reverse-transcribed using β-actin mRNA 3′-UTR-specific primers and AMV reverse transcriptase (Promega) for 1 h at 42°C [8]. After 25 cycles, the products were analyzed on 2% agarose gels.

### Polysome profiling analysis using sucrose gradients

Two independent p100 plates of subconfluent HeLa cells with control or TIA1 + TIAR reduced expression were used in this study. Five minutes before lysis, cells were incubated with 50 μg/ml of cycloheximide (CHX) to freeze the polysomes. Cells were washed twice with cold PBS-CHS and lysed in polysome buffer (Hepes 30 mM pH 7.4, 100 mM KCl, 5 mM MgCl_2_, 1 mM DTT, 1% Triton X-100) supplemented with 0.5 mg/ml of heparin and 50 μg/ml of cycloheximide. After 15 min of incubation, cells were centrifuged at 8,000×g in a microfuge at 4°C. Supernatant was immediately loaded on ultracentrifuge tubes containing a 15-40% sucrose gradient in polysome buffer. Gradient were spun at 39,000 rpm for 2 h in a SW40 rotor (Beckman) at 4°C. Tubes were fractionated from the top to get 11 fractions that were extracted immediately with phenol:chloroform and precipitated with sodium acetate, 2 volumes of ethanol and glycoblue (Ambion) overnight at -70°C. An aliquot (1/10) of the RNA content of each fraction RNA was analyzed by 2% agarose-TBE electrophoresis. Fractions from 1 to 7 (free plus monosome) and from 8 to 11 (polysome) were pooled and precipitated with LiCl overnight at -20°C. The RNA pellet was washed with 70% ethanol and resuspended in lysis buffer of RNeasy kit (Qiagen) and purified according to the manufacturer’s instructions.

### Immunoprecipitation of metabolically labeled proteins and immunoprecipitation-RT-PCR (IP-RT-PCR) analysis

Anti-β-actin, anti-α-tubulin or anti-IgG antibodies were added to 20 μl of cytoplasmic extracts from control (c) and TIA1/TIAR (1 + R)-knocked down HeLa cells incubated with 1 ml methionine-cysteine free DMEM supplemented with 5 μl Easy Tag™ EXPRESS ^35^S Protein Labeling mix, [^35^S] Met-Cys (11 mCi/ml, 37.0 Tbq/mmol; Perkin Elmer) for 30 min, complemented with buffer D to a total volume of 25 μl and incubated for 1 h on ice. After addition of 20 μl of protein G-Sepharose beads (1/1) and 40 μl of IPP 100 buffer (10 mM Tris pH 8.0, 100 mM NaCl, 0.1% NP-40), the reaction was incubated for 1 h on a rotating wheel at 4°C. The beads were collected at 1,000 rpm and washed four times with ice-cold IPP 100 buffer. A 2 μl aliquot of each sample was removed as loading control. The immunoprecipitated antibody-protein complexes were released from the agarose beads by boiling in Laemmli sample buffer and analyzed by 10% SDS–PAGE and autoradiography. For IP-RT-PCR assays, β-actin mRNA was coimmunoprecipitated from HeLa cell extracts prepared by the Lejeune and Maquat method [48] in NET-2 buffer (50 mM Tris–HCl, pH 7.4; 300 mM NaCl; 0.05% NP-40 plus a cocktail of protease inhibitors). Antibodies against TIA1, TIAR, HuR, U2AF65 or alone beads and 200 U of RNase inhibitor (Promega) were added to whole-cell extracts, and the samples incubated for 1 h at 4°C using end-over-end rotation. Protein G agarose beads (50 μl), previously incubated with yeast RNA for 1 h at 4°C, were added and the samples incubated for 1.5 h at 4°C and washed five times with 1 ml NET-2 buffer. RNA associated with the antibody–antigen complexes was eluted with sample buffer (0.1 M Tris–HCl, pH 6.8; 4% SDS; 12% β-mercaptoethanol; 20% glycerol), extracted with phenol:chloroform, precipitated with sodium acetate plus glycoBlue (Ambion) and ethanol, treated with DNase, and finally analyzed by semiquantitative RT-PCR.

### Poly (A) test (PAT) assays

Reverse transcription and polymerase chain reaction with PAT (RT-PCR-PAT), rapid amplification of cDNA ends PAT (RACE-PAT) and RNA-ligation coupled PAT (RLC-PAT) were carried out as described previously [49].

### Preparation of in vitro synthesized RNA probes, electrophoretic mobility shift assays (EMSA) and ultraviolet crosslinking (UV-CXL) analysis

*In vitro* transcription reactions were performed with T7 RNA polymerase from PCR template as described previously [46]. EMSA and UV crosslinking assays were carried out as previously described [46] using 100,000 cpm of ^32^P-labeled RNAs and the purified recombinant fusion proteins indicated in the Figure legends. The RNA-protein binding reaction was carried out in a total volume of 10 μl of buffer D containing 100 mM KCl and 3 μg of yeast tRNA. The ^32^P-labeled RNA-protein complexes were fractionated on 5% native PAGE gel for EMSA or 10% SDS–PAGE gels for UV-CXL and visualized by autoradiography.

## Abbreviations

ACTB: Actin beta subunit; ARE: AU-rich sequence element; CHX: Cycloheximide; EMSA: Electrophoretic mobility shift assay; GAPDH: Glyceraldehyde 3-phosphate dehydrogenase; GCN2: General control non-derepressible 2; GST: Glutathione-S-transferase; HuR/ELAVL1: Hu antigen R/embryonic lethal, abnormal vision, *Drosophila*-like (ELAVL); MBP: Maltose-binding protein; MEF: Murine embryonic fibroblast; mTOR: Mammalian target of rapamycin; MTT: Methyl thiazolyl tetrazolium; RNAi: RNA interference; PAT: Poly (A) test assay; RACE-PAT: Rapid amplification of cDNA ends PAT; RLC-PAT: RNA-ligation coupled PAT; RT-PCR-PAT: Reverse transcription and polymerase chain reaction with PAT; siRNA: Small interfering RNA; shRNA: Short hairpin RNA; TIA1: T-cell intracellular antigen 1; TIA-iCLIP: *In vivo* ultraviolet crosslinking and immunoprecipitation analysis of TIA1 and TIAR proteins; TIAR/TIAL1: TIA1 related/like protein; 5′-TOP: 5′-terminal oligopyrimide tract; 5′/3′-UTR: 5′ and 3′-untranslated regions of the mRNAs.

## Competing interests

The authors declare that they have no competing interests.

## Authors’ contributions

JMI conceived the research and designed all the experiments. IC, CSJ and JMI carried out the experiments presented in this paper. IC, CSJ and JMI wrote the paper. All authors provided feedback and approved the final manuscript.

## Supplementary Material

Additional file 1: Figure S1 RNAi-mediated knockdown on TIA and HuR proteins in HeLa cells. (A) HeLa cells were transfected with empty plasmid or plasmids expressing shRNAs against TIA1, TIAR or HuR mRNAs. The resulting stable HeLa cell lines were stained with anti-TIA1, anti-TIAR, anti-HuR and anti-α-tubulin antibodies. The fluorescence intensity of ΤιΑ1, TIAR, HuR and α-tubulin were quantified at least 30 different images per sample using ImageJ software. These quantifications are represented by histograms where the white, lightgray, darkgray, black and gray bars correspond to the control, TIA1, TIAR, TIA1 plus TIAR (1 + R) and HuR samples, respectively. The represented values were normalized and expressed relative to control (c), whose value is fixed arbitrarily to 1, and are means ± SEM (n = 30). The scale bar shows 8 μm. Click here for file

Additional file 2: Figure S2 Crosslinking sites (iCLIP) of TIA1 and TIAR proteins at 3′-UTR of the human β-actin mRNA. The RNA map corresponding to TIA proteins on β-actin pre-mRNA in HeLa cells was adapted using the TIA-iCLIP analysis provided by Jernej Ule’s laboratory [9]. The bar graphs show the number of cDNAs that identified each crosslinking site. The exon and intron positions of the human β-actin (ACTB) pre-mRNA are indicated. The full-picture of the TIA-iCLIP analysis on human β-actin 3′-UTR sequence is shown at the nucleotide level. Click here for file

Additional file 3: Figure S3 Characterization of the human 3′-UTR of β-actin mRNA by poly (A) test (PAT) assays. (A-C) Reverse transcription and polymerase chain reaction with PAT (RT-PCR-PAT) (A), rapid amplification of cDNA ends PAT (RACE-PAT) (B) and RNA-ligation coupled PAT (RLC-PAT) (C) are shown. These PAT assays were carried out as previously described [49]. The results suggest that 3′-UTR heterogeneity (size and polyadenylation degree) of human β-actin mRNA is similar in control and TIA1/TIAR (1 + R)-knocked down HeLa cells. Molecular weight markers for DNA are indicated on the left. The identities of DNA bands are indicated on the right by arrowheads. Click here for file

Additional file 4: Figure S4 Ectopic expression of GFP-tagged TIAR inhibits the translation of a chimeric GFP RNA containing human 3′-UTR. Western blot analysis of HeLa cell extracts (10 μg) prepared 24 h post-transfection with a control plasmid (c) or plasmids expressing GFP-tagged TIAR or HuR proteins together with either GFP or GFP-β-actin 3′-UTR reporter plasmids. The represented values by histograms were normalized and are expressed relative to control (c), whose value is fixed arbitrarily to 1, and are means ± SEM (from two independent analysis). Molecular weight markers for protein are indicated on the left. The identities of protein bands are indicated on the right by arrowheads. Click here for file

Additional file 5: Figure S5 Multiple alignment of 3′-UTR sequences of human and mouse β-actin mRNAs. The nucleotide sequences corresponding to the 3′ UTR of human and mouse β-actin mRNAs were aligned using ClustalW program. The NCBI nucleotide accession numbers of human and mouse β-actin mRNAs are indicated as follows: *Homo sapiens*: NM_001101.3 and *Mus musculus*: NM_007393.3. Observe the high degree of conservation of uridine-rich sequences downstream from stop codon. Conservation is also observed towards the center and 3′-end of the 3′-UTRs. Stop codons, U-rich sequence elements and polyadenylation signals are underlined and coloured. Click here for file

Additional file 6: Figure S6 Transient depletion of TIA proteins in HeLa cells did not alter β-actin protein expression. (A) Positions and sequences of the siRNAs used for RNA interference of TIA1 and TIAR. Location refers to positions of the first and last nucleotides in full-length cDNAs. As control siRNA was used a non-silencing siRNA duplex fluorescein labeled 27-6411-02FL from Gene Link. (B) Western blot analysis of HeLa cell extracts (2 μg (lane 1; c (1/5)) or 10 μg (lanes 2 to 4) prepared 72 h after transfection with siRNAs against control (c, lanes 1 and 2) and TIA1 plus TIAR (lanes 3 and 4; 1 + R). The transient knockdown of TIA1 and TIAR protein levels was approximately 70-80% under these conditions. The blot was probed with antibodies against TIA1, TIAR, HuR, β-actin and α-tubulin proteins, as indicated. Molecular weight markers and the identities of protein bands are shown. Click here for file
