# Supplementary figures and images for: Long-term reduction of T-cell intracellular antigens leads to increased beta-actin expression
Source: Mol Cancer. 2014 Apr 27;13:90. doi: 10.1186/1476-4598-13-90 (PMC4113145; doi:10.1186/1476-4598-13-90)

Fig. S1

A

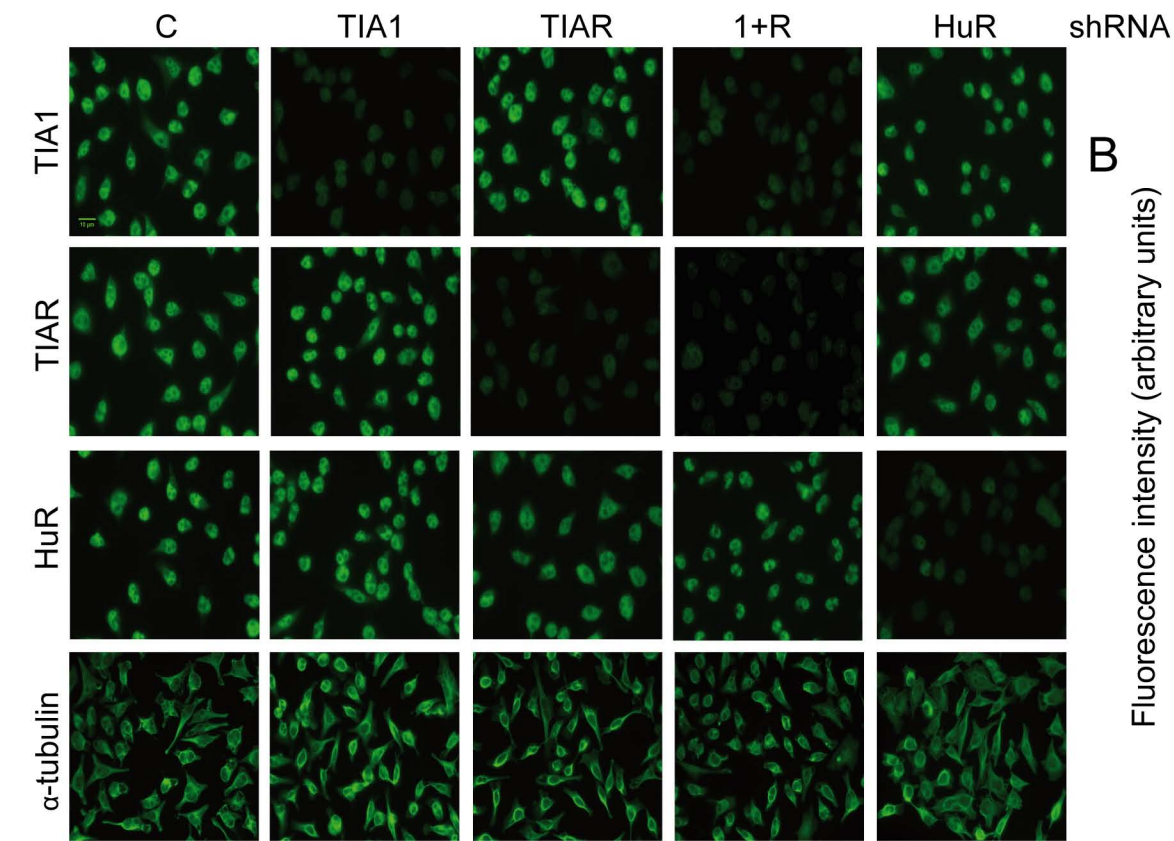

B

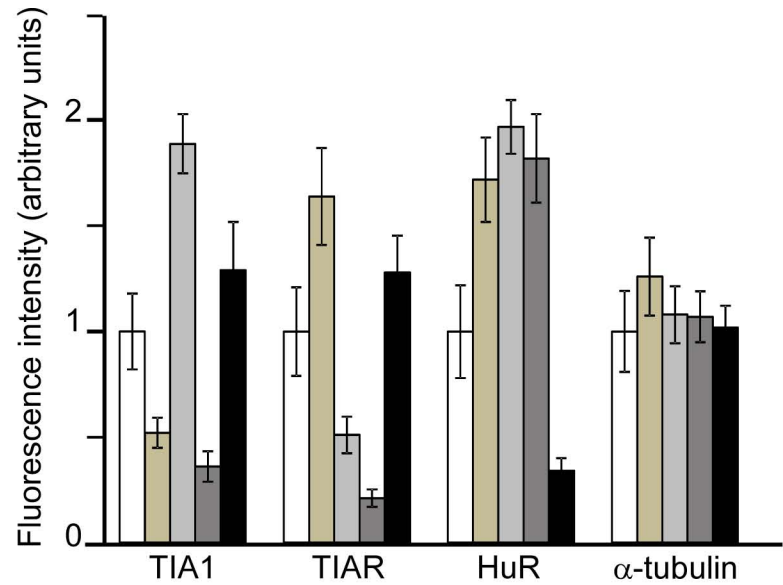

Supplement: Additional file 1: Figure S1 — RNAi-mediated knockdown on TIA and HuR proteins in HeLa cells. (A) HeLa cells were transfected with empty plasmid or plasmids expressing shRNAs against TIA1, TIAR or HuR mRNAs. The resulting stable HeLa cell lines were stained with anti-TIA1, anti-TIAR, anti-HuR and anti-α-tubulin antibodies. The fluorescence intensity of ΤιΑ1, TIAR, HuR and α-tubulin were quantified at least 30 different images per sample using ImageJ software. These quantifications are represented by histograms where the white, lightgray, darkgray, black and gray bars correspond to the control, TIA1, TIAR, TIA1 plus TIAR (1 + R) and HuR samples, respectively. The represented values were normalized and expressed relative to control (c), whose value is fixed arbitrarily to 1, and are means ± SEM (n = 30). The scale bar shows 8 μm. [file 1476-4598-13-90-S1.pdf]

Fig. S4

A

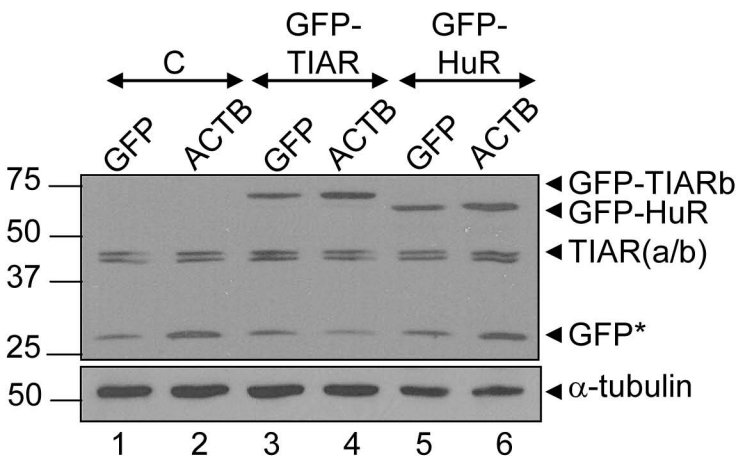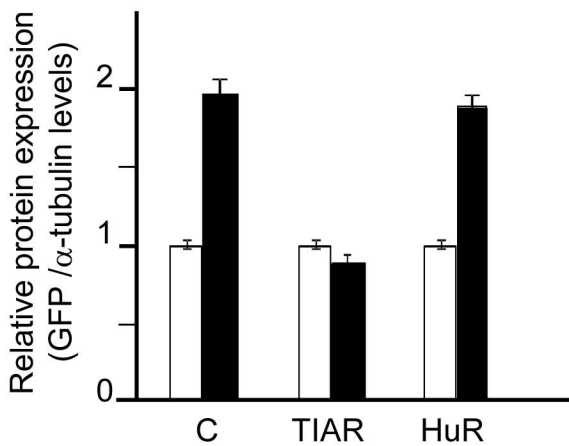

Supplement: Additional file 4: Figure S4 — Ectopic expression of GFP-tagged TIAR inhibits the translation of a chimeric GFP RNA containing human 3′-UTR. Western blot analysis of HeLa cell extracts (10 μg) prepared 24 h post-transfection with a control plasmid (c) or plasmids expressing GFP-tagged TIAR or HuR proteins together with either GFP or GFP-β-actin 3′-UTR reporter plasmids. The represented values by histograms were normalized and are expressed relative to control (c), whose value is fixed arbitrarily to 1, and are means ± SEM (from two independent analysis). Molecular weight markers for protein are indicated on the left. The identities of protein bands are indicated on the right by arrowheads. [file 1476-4598-13-90-S4.pdf]
