# Supplementary material for: Long-term reduction of T-cell intracellular antigens leads to increased beta-actin expression
Source: Mol Cancer. 2014 Apr 27;13:90. doi: 10.1186/1476-4598-13-90 (PMC4113145; doi:10.1186/1476-4598-13-90)

# Fig. S2

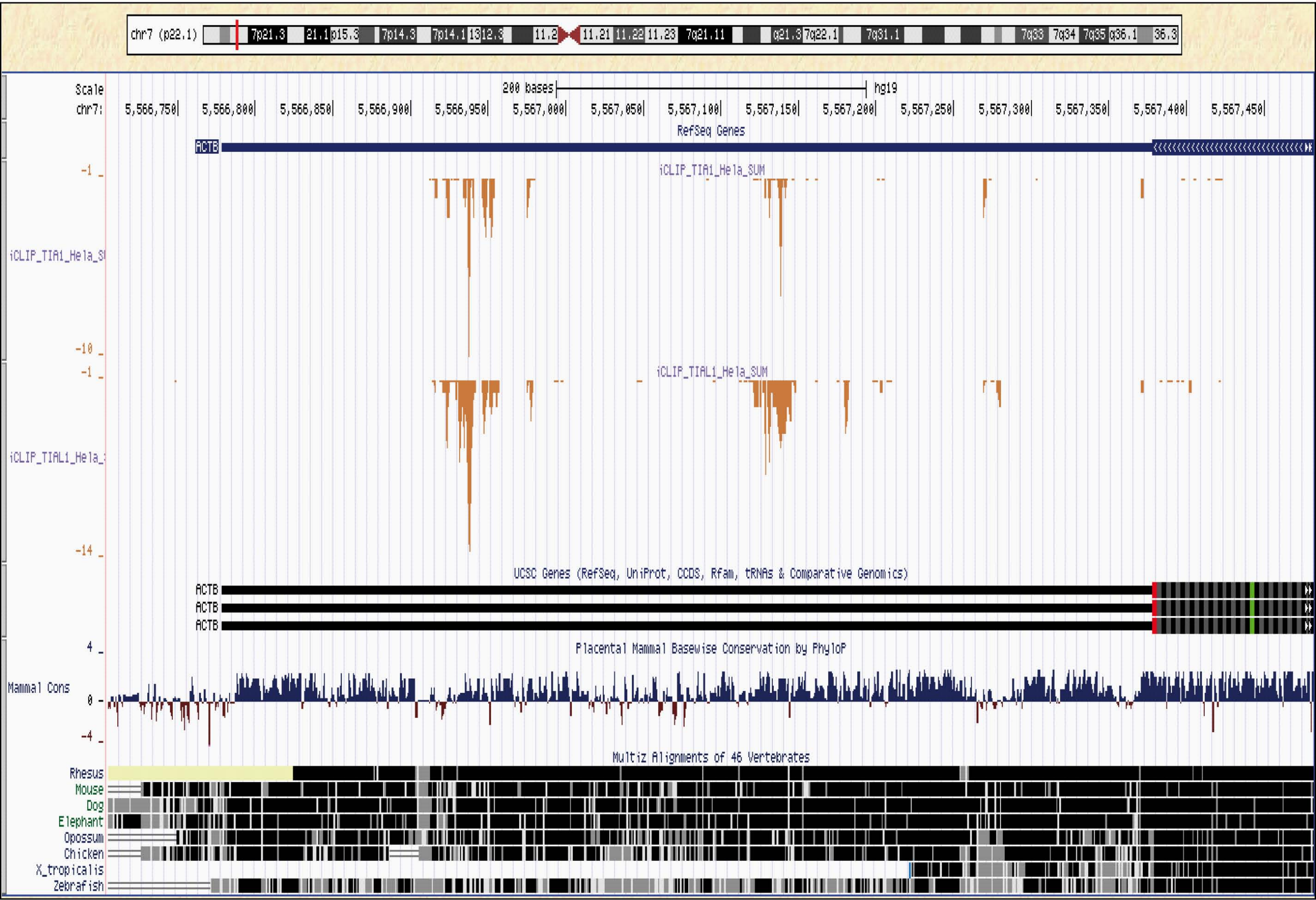

chr7 (p22.1)
7p21.3
21.1
p15.3
7p14.3
7p14.1
13
12.3
11.2
11.21
11.22
11.23
7q21.11
q21.3
7q22.1
7q31.1
7q33
7q34
7q35
q36.1
36.3
Scale
200 bases
hg19
chr7:
5,566,750
5,566,800
5,566,850
5,566,900
5,566,950
5,567,000
5,567,050
5,567,100
5,567,150
5,567,200
5,567,250
5,567,300
5,567,350
5,567,400
5,567,450
RefSeq Genes
ACTB
iCLIP_TIA1_Hela_SUM
-1
-10
iCLIP_TIAL1_Hela_SUM
-1
-14
UCSC Genes (RefSeq, UniProt, CCDS, Rfam, tRNAs & Comparative Genomics)
ACTB
ACTB
ACTB
Placental Mammal Basewise Conservation by PhyloP
Mammal Cons
4
0
-4
Multiz Alignments of 46 Vertebrates
Rhesus
Mouse
Dog
Elephant
Opossum
Chicken
X_tropicalis
Zebrafish

# *iCLIP* of TIA1/TIAR against ACTB mRNA 3'-UTR (Full-picture, I)

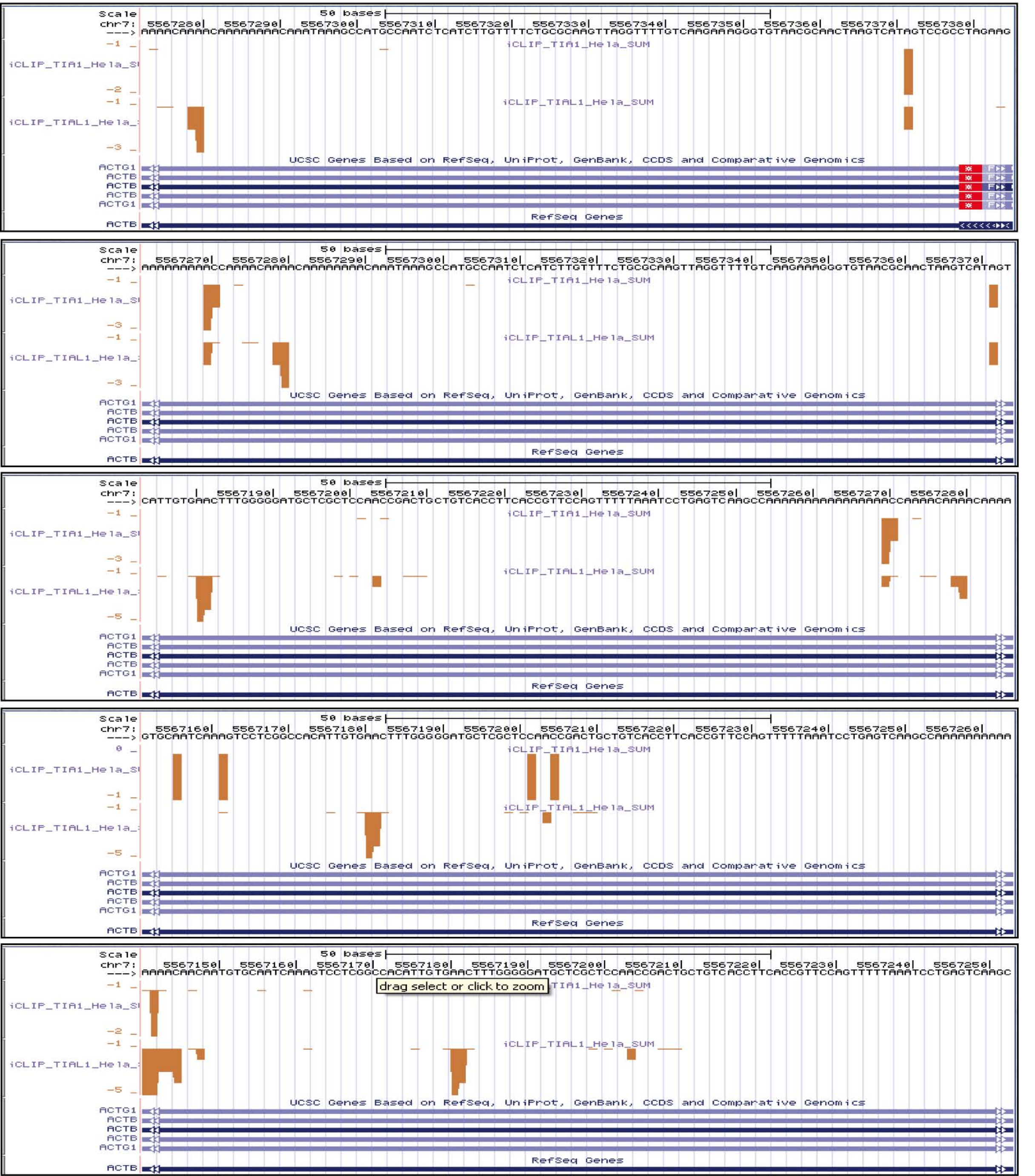

# *iCLIP* of TIA1/TIAR against ACTB mRNA 3’-UTR (Full-picture, II)

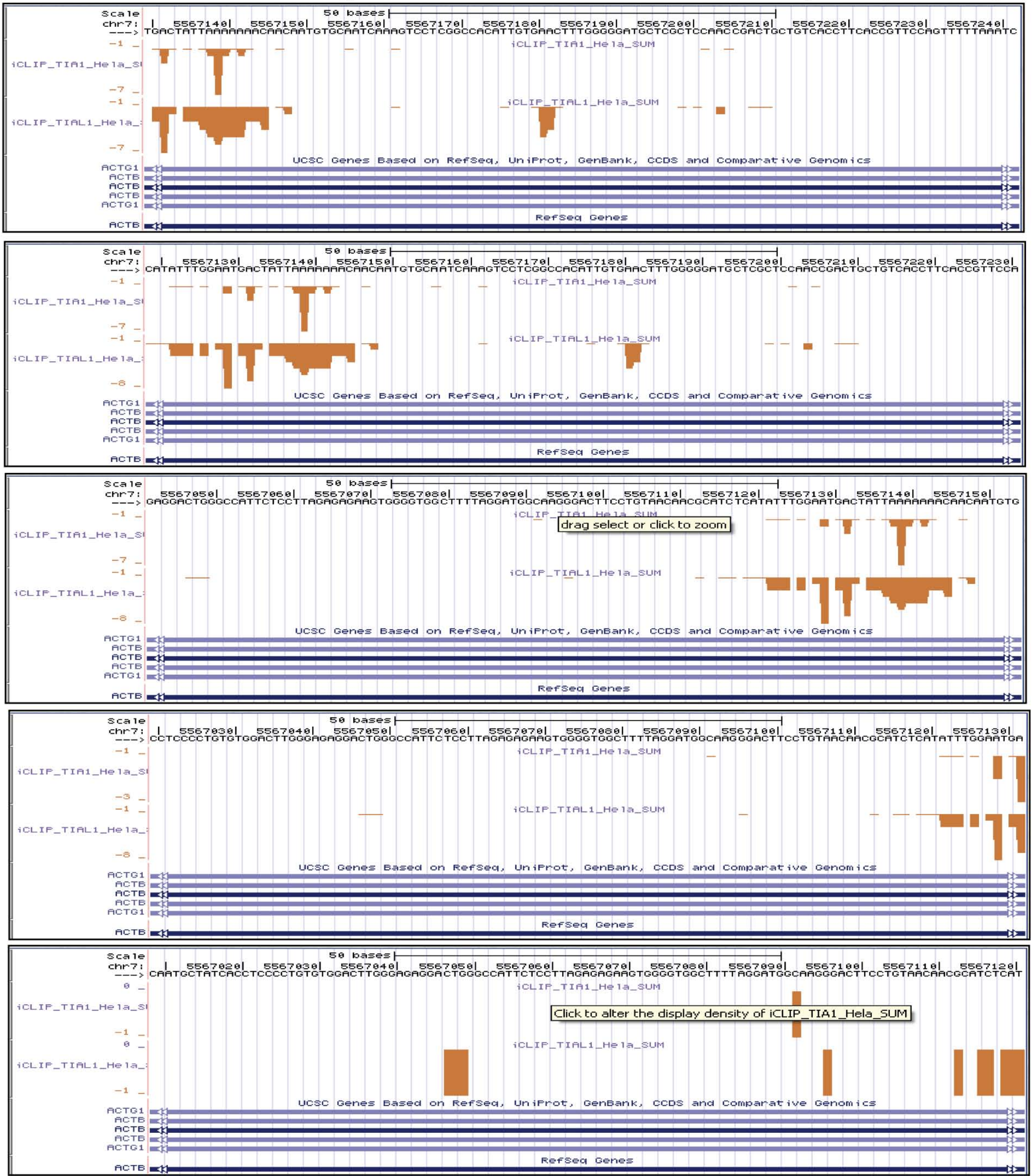

# *iCLIP* of TIA1/TIAR against ACTB mRNA 3'-UTR (Full-picture, III)

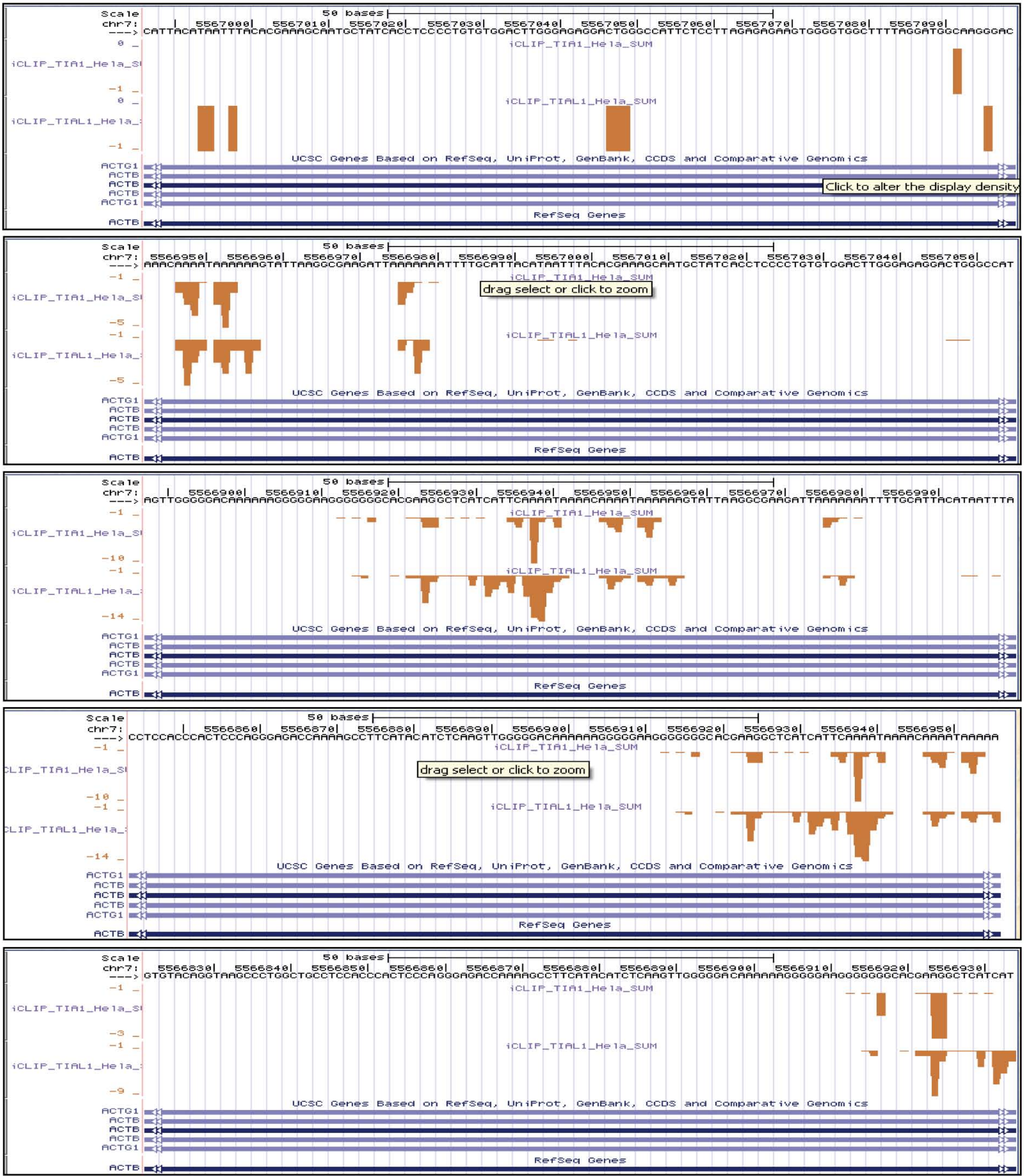

# *iCLIP* of TIA1/TIAR against ACTB mRNA 3'-UTR (Full-picture, IV)

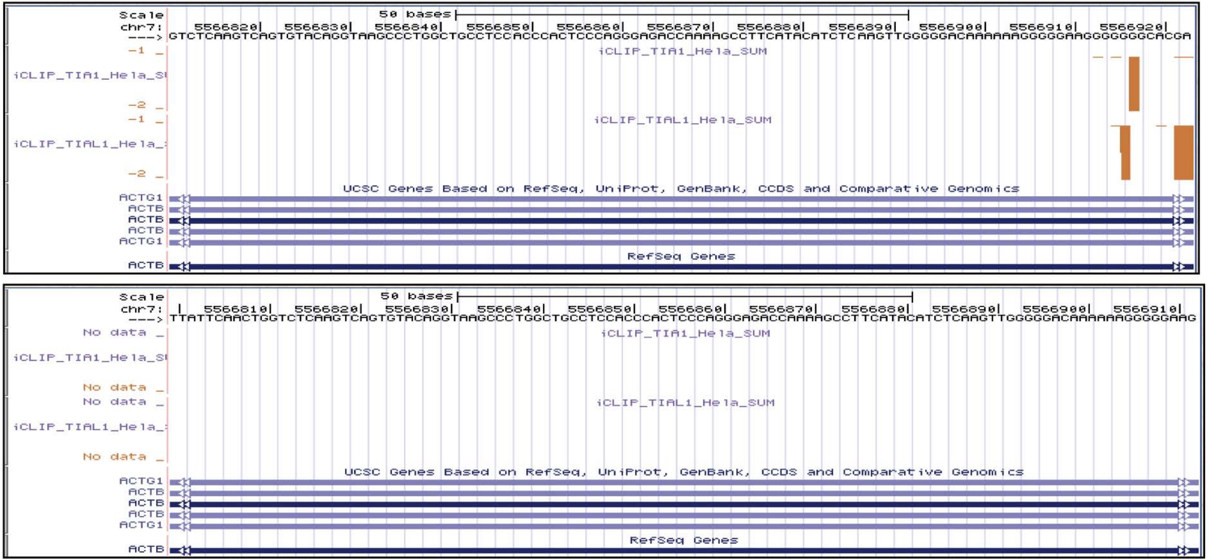

Supplement: Additional file 2: Figure S2 — Crosslinking sites (iCLIP) of TIA1 and TIAR proteins at 3′-UTR of the human β-actin mRNA. The RNA map corresponding to TIA proteins on β-actin pre-mRNA in HeLa cells was adapted using the TIA-iCLIP analysis provided by Jernej Ule’s laboratory [9]. The bar graphs show the number of cDNAs that identified each crosslinking site. The exon and intron positions of the human β-actin (ACTB) pre-mRNA are indicated. The full-picture of the TIA-iCLIP analysis on human β-actin 3′-UTR sequence is shown at the nucleotide level. [file 1476-4598-13-90-S2.pdf]
