# Supplementary material for: Long-term reduction of T-cell intracellular antigens leads to increased beta-actin expression
Source: Mol Cancer. 2014 Apr 27;13:90. doi: 10.1186/1476-4598-13-90 (PMC4113145; doi:10.1186/1476-4598-13-90)
Supplement: Additional file 3: Figure S3 — Characterization of the human 3′-UTR of β-actin mRNA by poly (A) test (PAT) assays. (A-C) Reverse transcription and polymerase chain reaction with PAT (RT-PCR-PAT) (A), rapid amplification of cDNA ends PAT (RACE-PAT) (B) and RNA-ligation coupled PAT (RLC-PAT) (C) are shown. These PAT assays were carried out as previously described [49]. The results suggest that 3′-UTR heterogeneity (size and polyadenylation degree) of human β-actin mRNA is similar in control and TIA1/TIAR (1 + R)-knocked down HeLa cells. Molecular weight markers for DNA are indicated on the left. The identities of DNA bands are indicated on the right by arrowheads. [file 1476-4598-13-90-S3.pdf]

Fig. S3

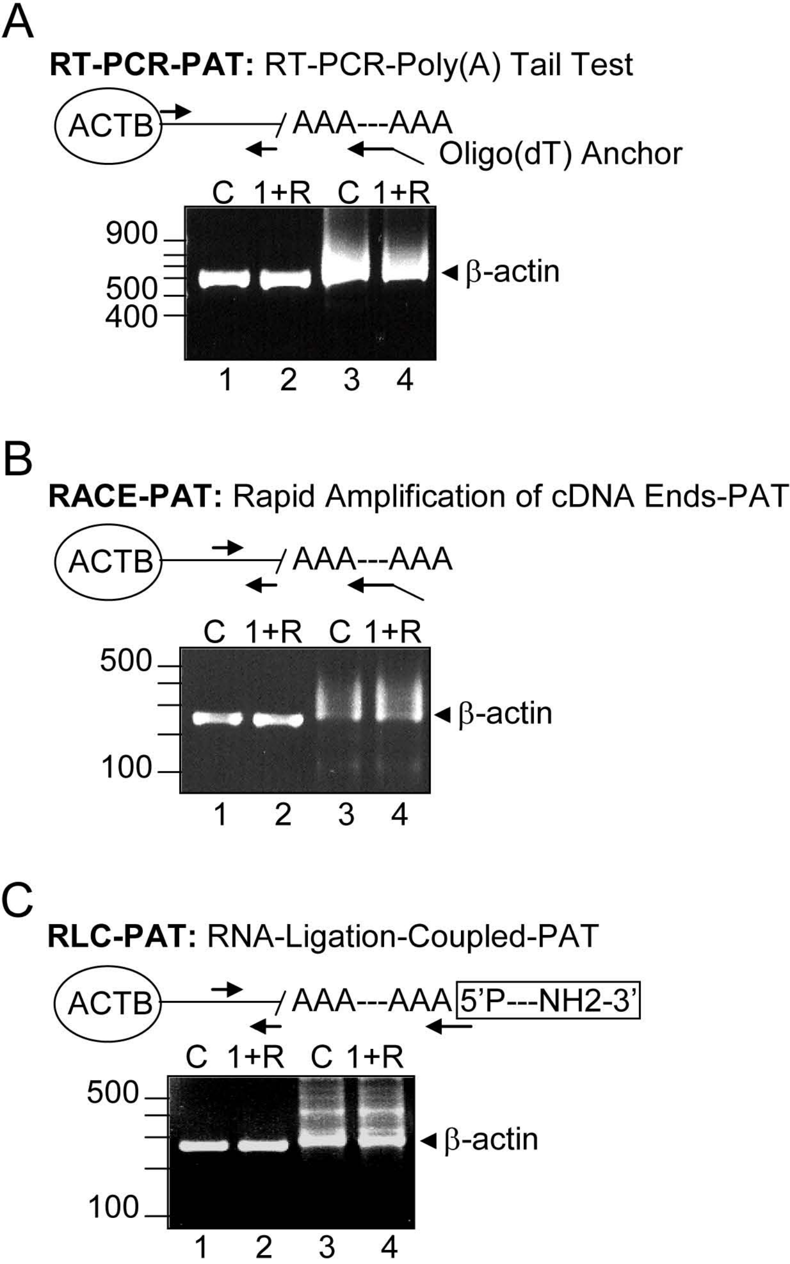
A
RT-PCR-PAT: RT-PCR-Poly(A) Tail Test
ACTB
AAA---AAA
Oligo(dT) Anchor
C 1+R C 1+R
900
500
400
◄ β-actin
1 2 3 4
B
RACE-PAT: Rapid Amplification of cDNA Ends-PAT
ACTB
AAA---AAA
C 1+R C 1+R
500
100
◄ β-actin
1 2 3 4
C
RLC-PAT: RNA-Ligation-Coupled-PAT
ACTB
AAA---AAA
5'P---NH2-3'
C 1+R C 1+R
500
100
◄ β-actin
1 2 3 4
