# Supplementary material for: Long-term reduction of T-cell intracellular antigens leads to increased beta-actin expression
Source: Mol Cancer. 2014 Apr 27;13:90. doi: 10.1186/1476-4598-13-90 (PMC4113145; doi:10.1186/1476-4598-13-90)
Supplement: Additional file 5: Figure S5 — Multiple alignment of 3′-UTR sequences of human and mouse β-actin mRNAs. The nucleotide sequences corresponding to the 3′ UTR of human and mouse β-actin mRNAs were aligned using ClustalW program. The NCBI nucleotide accession numbers of human and mouse β-actin mRNAs are indicated as follows: Homo sapiens: NM_001101.3 and Mus musculus: NM_007393.3. Observe the high degree of conservation of uridine-rich sequences downstream from stop codon. Conservation is also observed towards the center and 3′-end of the 3′-UTRs. Stop codons, U-rich sequence elements and polyadenylation signals are underlined and coloured. [file 1476-4598-13-90-S5.pdf]

Fig. S5

```
Human  NM_001101.3 1198 CGCAAATGCTTCTAGGCGGACTATGACTTAGTTGCG--TTACACCCTTTC 1245
                        |||||.||||||||||||||||.|.|||.||.||||  ||||||||||||
Mouse  NM_007393.3 1193 CGCAAGTGCTTCTAGGCGGACTGTTACTGAGCTGCGTTTTACACCCTTTC 1242
       NM_001101.3 1246 -TTGACAAAACCTAACTTGCGCAGAAAACA----------AGATGAGATT 1284
                         |||||||||||||||||||||||||||.|          |||..|.|||
       NM_007393.3 1243 TTTGACAAAACCTAACTTGCGCAGAAAAAAAAAAAATAAGAGACAACATT 1292
       NM_001101.3 1285 GGCATGGCTTTATTT------------------GTTTTTTTTGTTTTGTT 1316
                        |||||||||||.|||                  |||||||||.||||.||
       NM_007393.3 1293 GGCATGGCTTTGTTTTTTTAAATTTTTTTTAAAGTTTTTTTTTTTTTTTT 1342
       NM_001101.3 1317 TT-------------GGTTTTTTTTTTTTTTTTGGC----TTGACTCAGG 1349
                        ||             |.||||||.||||.|||||||    ||||||||||
       NM_007393.3 1343 TTTTTTTTTTTTTAAGTTTTTTTGTTTTGTTTTGGCGCTTTTGACTCAGG 1392
       NM_001101.3 1350 ATTTAAAAACTGGAACGGTGAAGGTGACAGCAGTCGGTTGGAGCGAGCAT 1399
                        ||||||||||||||||||||||||.|||||||||.|||||||||.|.|||
       NM_007393.3 1393 ATTTAAAAACTGGAACGGTGAAGGCGACAGCAGTTGGTTGGAGCAAACAT 1442
       NM_001101.3 1400 CCCCCAAAGTTC-AC-AATGTGGCCGAGGACTTTGATTGCACATTGT--- 1444
                        |||||||||||| || ||||||||.|||||||    |||.|||||||
       NM_007393.3 1443 CCCCCAAAGTTCTACAAATGTGGCTGAGGACT----TTGTACATTGTTTT 1488
       NM_001101.3 1445 -----------------------TGTTTTTTTAATAGTCATTCCAAAT-- 1469
                                               |.|||||||||||||||||||||.|
       NM_007393.3 1489 GTTTTTTTTTTTTTTTGGTTTTGTCTTTTTTTAATAGTCATTCCAAGTAT 1538
       NM_001101.3 1470 --ATGAGATGCGTTGTTACAGGAAGTCCCTTGCCATCCTAAAAGCCA-CC 1516
                          ||||.||..||.||||||||||||||||..||.|||.|||||||| ||
       NM_007393.3 1539 CCATGAAATAAGTGGTTACAGGAAGTCCCTCACCCTCCCAAAAGCCACCC 1588
       NM_001101.3 1517 CCACTTCTCTCTA--AGGAGAATGGCCCAGTCC-TCTCCCAAGTCCACAC 1563
                        |||||    .|||  |||||.||||.|..|||| |..||..|||||||.|
       NM_007393.3 1589 CCACT----CCTAAGAGGAGGATGGTCGCGTCCATGCCCTGAGTCCACCC 1634
       NM_001101.3 1564 AGGGG-AGGTGATAGCATTGCTTTCGTGTAAATTATGTAATGCAAAA--T 1610
                        .|||| ||||||.||||||||||..||||||||||||||.|||||||  |
       NM_007393.3 1635 CGGGGAAGGTGACAGCATTGCTTCTGTGTAAATTATGTACTGCAAAAATT 1684
       NM_001101.3 1611 TTTTTTAATCTT-CGCCTTAATACTTTTTTATTTTGTTTT--ATTT-TGA 1656
                        |||||.|||||| |||||||||||||..|  |||||||||  |||| |||
       NM_007393.3 1685 TTTTTAAATCTTCCGCCTTAATACTTCAT--TTTTGTTTTTAATTTCTGA 1732
       NM_001101.3 1657 ATGATGAGCC----TTCGTGCCCCCCCTTCCCCCTTTTTTGT-CCCCCAA 1701
                        |||    |||    |..|.|.||.|||||     |||||||| |||||||
       NM_007393.3 1733 ATG----GCCCAGGTCTGAGGCCTCCCTT-----TTTTTTGTCCCCCCAA 1773
       NM_001101.3 1702 CTTGAGATGTATGAAGGCTTTTGGTCTCCCTGGGAGTGG--------GTG 1743
                        |||  ||||||||||||| |||||||||||||||||.||        ||.
       NM_007393.3 1774 CTT--GATGTATGAAGGC-TTTGGTCTCCCTGGGAGGGGGTTGAGGTGTT 1820
       NM_001101.3 1744 GAGGCAGCCAGGGCTTACCTGTACACTGACTTGAGACCAGTTGAATAAAA 1793
                        |||||||||||||||..|||||||||||||||||||||     |||||||
       NM_007393.3 1821 GAGGCAGCCAGGGCTGGCCTGTACACTGACTTGAGACC-----AATAAAA 1865
       NM_001101.3 1794 GTGCACACCTTA--------AAAATGA 1812
                        ||||||||||||        |||.
       NM_007393.3 1866 GTGCACACCTTACCTTACACAAAC--- 1889
```
