# Supplementary material for: Long-term reduction of T-cell intracellular antigens leads to increased beta-actin expression
Source: Mol Cancer. 2014 Apr 27;13:90. doi: 10.1186/1476-4598-13-90 (PMC4113145; doi:10.1186/1476-4598-13-90)

Fig. S6

A

Name	Position	cDNA Sequence	Target
C	-	non-silencing siRNA duplex	-
1	647-669	5'-AACAACTAATGCGTCAGACTTTT-3'	TIA1
R	65-87	5'-AAGTCCTTATACTTCAGTTGTTC-3'	TIAR

B

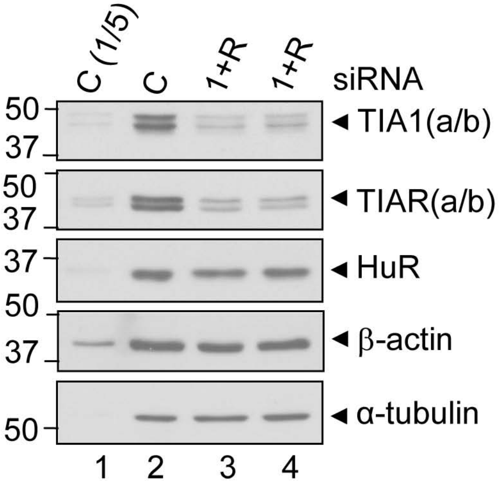

Supplement: Additional file 6: Figure S6 — Transient depletion of TIA proteins in HeLa cells did not alter β-actin protein expression. (A) Positions and sequences of the siRNAs used for RNA interference of TIA1 and TIAR. Location refers to positions of the first and last nucleotides in full-length cDNAs. As control siRNA was used a non-silencing siRNA duplex fluorescein labeled 27-6411-02FL from Gene Link. (B) Western blot analysis of HeLa cell extracts (2 μg (lane 1; c (1/5)) or 10 μg (lanes 2 to 4) prepared 72 h after transfection with siRNAs against control (c, lanes 1 and 2) and TIA1 plus TIAR (lanes 3 and 4; 1 + R). The transient knockdown of TIA1 and TIAR protein levels was approximately 70-80% under these conditions. The blot was probed with antibodies against TIA1, TIAR, HuR, β-actin and α-tubulin proteins, as indicated. Molecular weight markers and the identities of protein bands are shown. [file 1476-4598-13-90-S6.pdf]
